# Polarization-dependent gain characterization in x-cut LNOI erbium-doped waveguide amplifiers

**DOI:** 10.1515/nanoph-2025-0335

**Published:** 2025-11-25

**Authors:** Jiayu Huang, Run Li, Suo Wang, Qianqian Jia, Zichuan Xiang, Jinling Yang, Jinye Li, Jianguo Liu

**Affiliations:** Laboratory of Nano Optoelectronics, Institute of Semiconductors, 71243 Chinese Academy of Sciences, Beijing 100083, China; College of Materials Science and Opto-Electronic Technology, University of Chinese Academy of Sciences, Beijing 100049, China; Institute of Semiconductors, Chinese Academy of Sciences, Beijing 100083, China; Institute for Intelligent Photonics, Nankai University, Tianjin 300071, China

**Keywords:** erbium-doped waveguide amplifier, x-cut lithium niobate on insulator, polarization-dependent gain, integrated photonics

## Abstract

Erbium-doped waveguide amplifiers (EDWAs) are vital for photonic integration, yet most are built on z-cut lithium niobate, incompatible with the mainstream x-cut platform. This work presents a combined theoretical and experimental study of polarization-dependent gain in x-cut Er:LNOI. Using Judd–Ofelt theory, we analyze how crystal orientation governs TE-mode coupling to Er^3+^ ions, predicting stark differences in transition strengths between *α*- and *π*-polarizations. Experiments confirm these predictions: at 1,531 nm, the absorption and emission cross sections for *α*-polarization are 1.8 times larger than for *π*-polarization. At 1,550 nm, the *α*-polarization shows a gain coefficient of 3.3 dB/cm versus 2.2 dB/cm for *π*-polarization. In the small-signal regime, the *α*-polarized amplifier achieves 32.01 dB signal enhancement with 11.18 dB internal net gain. With 9.1 dBm on-chip input power, it delivers 21.18 mW unsaturated output power under pumping levels exceeding 200 mW. This work demonstrates feasible optical amplification on x-cut LNOI, providing crucial support for large-scale photonic and microwave photonic systems.

## 1 Introduction

As a key component in modern optical communication systems, erbium-doped fiber amplifiers (EDFAs) have provided essential technological support for the development of global ultra-broadband fiber networks, owing to their high gain, low noise, and excellent compatibility [1]. However, with the rapid advancement of integrated photonics, on-chip active devices are playing an increasingly important role. Among them, on-chip optical amplifiers are a critical element for realizing large-scale integration of active and passive photonic circuits. Building on the success of EDFAs, EDWAs offer effective chip-scale gain to overcome signal attenuation in on-chip optical interconnects [2], making them indispensable for realizing high-performance integrated photonic systems of the future.

Over the past few decades, EDWAs have been extensively investigated in various thin film materials, including silicon nitride [2], [3], [4], aluminum oxide [5], tellurium oxide [6], silicate [7], etc. However, achieving compact EDWAs with high gain remains challenging on these platforms. Recently, LNOI has emerged as a promising platform for multifunctional integrated photonic circuits due to its wide transparent window, excellent electro-optic properties, high nonlinear coefficient, and compatibility with micro–nano fabrication [8], [9], [10], [11]. A wide range of high-performance devices based on LNOI have been demonstrated, such as high-speed electro-optic modulators [12], [13], [14], [15], [16], optical frequency combs [17], [18], [19], efficient wavelength converters [20], [21], and on-chip spectrometers [22], providing advances in large-scale optical interconnects, optical computing, and quantum information processing. Against this backdrop, developing erbium-doped lithium niobate waveguide amplifiers compatible with the LNOI platform would not only enable efficient on-chip signal amplification but also pave the way for the monolithic integration of laser, modulation, and amplification functionalities, which is crucial for future low-power, high-bandwidth-density photonic computing and communication chips.

Several studies have reported EDWAs based on Er:LNOI [23], [24], [25], [26], [27], [28], [29], specifically, Chen et al. has achieved 5.2 dB small signal internal net gain at 1,530 nm [23], Liang et al. has achieved 20 dB small signal internal net gain at 1,532 nm [26], and Cai et al. has reported an output power of 16.65 dBm with 7.65 dB internal net gain at 1,550 nm [29]. These results highlight the potential of this platform for integrating active devices; however, they are all based on z-cut LNOI, whereas x-cut LNOI remains the mainstream choice for most photonic components. In particular, high-speed modulators and nonlinear converters rely on exploiting the strongest electro-optic coefficient (r33) along the *x*-axis. The inherent differences in crystal orientation between z-cut and x-cut LNOI fundamentally limit seamless integration, creating a pivotal bottleneck for large-scale active photonic systems. Therefore, developing EDWAs base on x-cut LNOI is essential to bridge this technological gap and enable fully integrated photonic circuits.

Here, we focus on x-cut LNOI and systematically investigate the polarization-dependent gain characteristics of erbium-doped lithium niobate waveguide amplifiers. Based on Judd–Ofelt theory, we analyze the variations in Er^3+^ transition strength and rate-equation parameters for different polarizations and experimentally verify their influence on amplifier performance. This study fills a critical gap in polarization-dependent amplification for x-cut Er:LNOI and provides new insight for cointegrating amplifiers and modulators on a unified LNOI platform, paving the way for high-performance active photonic circuits.

## 2 Polarization-resolved Judd–Ofelt analysis in x-cut Er:LNOI amplifier

The optical transitions of rare-earth ions in doped crystals are typically analyzed using the Judd–Ofelt (J–O) theory [30], [31]. This semi-empirical model enables the extraction of the J–O intensity parameters (Ω_2_, Ω_4_, Ω_6_) from polarization-resolved absorption spectra, allowing for the calculation of transition strengths between arbitrary energy levels of a given dopant–host system. These parameters are essential for predicting key laser and amplifier performance metrics, such as radiative transition probabilities, radiative lifetimes, and branching ratios.

**Figure 1:**
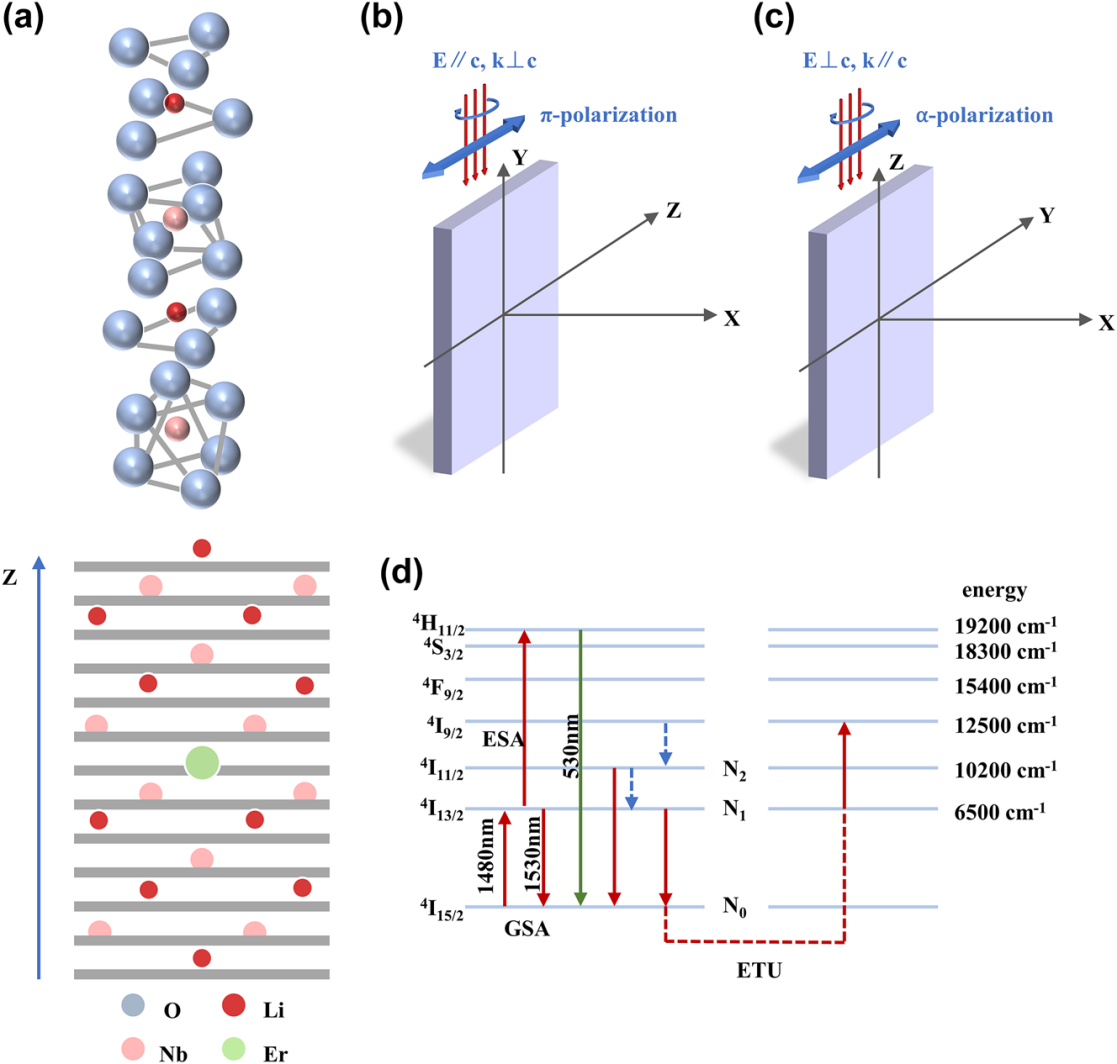
Structural and polarization characteristics of x-cut Er^3+^-doped LN and its relevant energy-level transitions. (a) Crystal structure of undoped LN (left) and a diagonal cut through the hexagonal unit cell of Er^3+^-doped LN, illustrating the possible substitutional positions of Er^3+^. (b) *π*-polarization, light propagates along the *y*-axis with the electric field oriented parallel to the optical axis (*c*-axis). (c) *α*-polarization, light propagates along the *z*-axis with the electric field oriented perpendicular to the optical axis. (d) Energy model of Er^3+^ under 1,480 nm pumping.

Lithium niobate (LN) belongs to the trigonal crystal system with space group *R*3*c*, and its optical axis typically lies along the *z*-axis (also referred to as the *c*-axis). In the undoped structure, both Li^+^ and Nb^5+^ ions are coordinated by six oxygen ions, forming distorted octahedral units, as illustrated in the upper panel of Figure 1(a). Upon erbium doping, most Er^3+^ ions substitute Li^+^ lattice sites or occupy nearby interstitial positions, while a smaller fraction may incorporate into Nb^5+^, NbLi^4+^, and defect cluster positions and so on [32], [33], as shown in the lower panel of Figure 1(a).

Due to the anisotropy of LN, its optical properties depend on the relative orientation between the electric field direction and the optical axis. When the electric field is perpendicular to the c-axis, the light propagates as an ordinary ray with refractive index *n*_0_; when it is parallel, the light behaves as an extraordinary ray with refractive index *n*_*e*_ [32], [34]. This anisotropy leads to different propagation constants and field distributions under distinct polarizations, which directly affect the coupling efficiency between the external field and the transition dipole of Er^3+^ ions.

It has been experimentally demonstrated that Er^3+^:LiNbO_3_ exhibits strongly polarization-dependent absorption spectra [34], with distinct features for *α*-polarization (*k*‖*c*), *σ*-polarization (*k*⊥*c*, *E*⊥*c*), and *π*-polarization (*k*⊥*c*, *E*‖*c*).

Within the J–O framework, the electric-dipole line strength (*S*_*q*,ed_) and magnetic-dipole line strength (*S*_*q*,md_) from an initial level *a* to *a* final level *b* under different polarization states are as follows [30], [31]

(1) $$S_{q,ed}\left(a,b\right)=e^{2}\underset{t=2,4,6}{\sum }\Omega_{t,q}{\left|\left\langle a\left\|U^{\left(t\right)}\right\|b\right\rangle \right|}^{2}$$

(2) $$S_{q,md}\left(a,b\right)={\left(\frac{eh}{4\pi mc}\right)}^{2}{\left|\left\langle a\left\|L,+,2,S\right\|b\right\rangle \right|}^{2}$$

where *e* is the electronic charge, Ω_*t*,*q*_ is the J–O parameters, ${\left\langle a\left\|U^{\left(t\right)}\right\|b\right\rangle }^{2}$ are double reduced matrix element of the tensor operator $U^{\left(t\right)}$ , *h* is the Planck’s constant, *m* is the mass of an electron, *c* is the speed of light in vacuum, and *L* + 2*S* is the md operator. Both ${\left\langle a\left\|U^{\left(t\right)}\right\|b\right\rangle }^{2}$ and *L* + 2*S* are independent of the host material. The subscript *q* denotes the polarization state of the transition, with *q* = *α*, *σ*, and *π*.

The theoretical transition strength from level *a* to *b* can be expressed by [31]

(3) $$f_{q,th}\left(a,b\right)=\frac{8\pi^{2}mc}{3h\left(2J_{a}+1\right)\bar{\lambda }}\left(X_{q,ed}\frac{S_{q,ed}}{e^{2}}+X_{q,md}\frac{S_{q,md}}{e^{2}}\right)$$

where $\bar{\lambda }$ is the mean wavelength of the transition between the two manifolds, and *J*_*a*_ is the total angular momentum of level *a*. The degeneracy of the level is defined by 2*J*_*a*_ + 1. *X*_*q*,ed_ and *X*_*q*,md_ are the electric-dipole and magnetic-dipole local field correction, respectively. According to the refractive index *n*_*q*_ under different polarization states, they can be expressed in terms of *n*_*q*_ as follows

(4) $$X_{ed}=\frac{{\left(n_{q}{\left(\lambda \right)}^{2}+2\right)}^{2}}{9n_{q}\left(\lambda \right)}$$

(5) $$X_{md}=n_{q}\left(\lambda \right)$$

By measuring the absorption spectra for different polarization states, the experimental transition strengths can be obtained using the expression [31]

(6) $$f_{q,meas}\left(a,b\right)=\frac{4m\varepsilon_{0}c^{2}}{Ne^{2}d{\left(\bar{\lambda }\right)}^{2}}\int \ln\left(10\right)\cdot OD{\left(\lambda \right)}_{i}d\lambda$$

where *N* is the number of dopant ions per unit volume, OD(*λ*)_*i*_ is the measure optical density under different polarization, defined as $\left[-\log_{10}T{\left(\lambda \right)}_{i}\right]$ , where T is the transmission through the material, and d is the thickness of the material.

The Ω_*t*,*q*_ are then determined by least-squares fitting to the theoretical transition strengths, and the strength of any radiative transition can be calculated.

According to the Ladenburg–Fuchtbauer (L–F) relation [33], the emission cross section (*σ*_em,*q*_) can be directly expressed as a function of the transition strength

(7) $$\sigma_{em,q}\left(\lambda \right)=\frac{e^{2}\lambda^{4}}{4\varepsilon_{0}mc^{2}{\left(\bar{\lambda }\right)}^{2}}f_{q,calu}\left(a,b\right)\Phi \left(\lambda \right)$$

where Φ(*λ*) is the normalized emission line-shape, and *λ* is the emission wavelength of interest.

From the above relationship, it is evident that the emission cross section is proportional to the transition strength. Since the J–O parameters Ω_*t*,*q*_ are polarization dependent, the electric dipole line strengths differ under different polarizations. In addition, variations in refractive indices lead to changes in the local field correction factors, further modifying the transition strength. As a result, the emission cross section exhibits a clear polarization dependence. According to the McCumber relation [35], the absorption cross section is also polarization dependent.

For x-cut Er:LNOI, the polarization state of the TE mode depends on the propagation direction of the waveguide, as the optical axis lies in the plane of the wafer plane. This is in contrast to z-cut films, where the TE mode always corresponds to a single polarization state (*σ*-polarization). As illustrated in Figure 1(b) and (c), when the waveguide propagates along the *z*-axis, the TE mode electric field is perpendicular to the optical axis, corresponding to *α*-polarization. Conversely, when the waveguide propagates along the *y*-axis, the TE mode electric field is parallel to the optical axis, corresponding to *π*-polarization.

Under 1,480 nm pumping, the gain dynamics of Er^3+^-doped waveguide amplifiers (EDWAs) on lithium niobate on insulator (LNOI) can be described using a steady-state three-level rate equation model [29], as shown in Figure 1(d). Based on the steady-state rate equations, both the net stimulated absorption rate of the pump light and the net stimulated emission rate of the signal light are directly proportional to the corresponding absorption and emission cross sections. Thus, the anisotropy of the J–O parameters directly gives rise to polarization-dependent amplifier gain.

Furthermore, we calculated the transition strength using the J–O parameters reported in Ref. [30] for different polarizations. Values of the host-independent matrix elements as tabulated for Er^3+^ ions in Ref. [36] were used, and the refractive indices have been obtained from the Sellmeier equation for LiNbO_3_ reported in the literature [37]. The EDWAs primarily involve the transition between the ^4^I_15/2_ and ^4^I_13/2_ energy levels under 1,480 nm pumping. The transition strength comprises both electric dipole and magnetic dipole components. Based on Eqs. (1)– (5), the calculation results are shown in Table 1. It should be noted that, due to experimental limitations, we were unable to measure multiple absorption bands to extract J–O parameters in this work, and thus the parameters reported in the literature [30] were used as references. Moreover, since both *α* and *σ* polarizations correspond to ordinary rays with refractive index *n*_0_, their J–O parameters can be considered approximately equivalent; therefore, the *σ*-polarization J–O parameters from the literature were adopted as a proxy for the *α*-polarization.

**Table 1:** Calculated transition strengths for different polarizations. All values are in units of 10^−6^.

Transition	*α*-polarization	*π*-polarization
^4^I_15/2_ → ^4^I_13/2_	1.36	1.15

From the calculation results, the transition strength of the *α*-polarization is larger than that of the *π*-polarization, corresponding to larger absorption and emission cross sections. Therefore, the amplification performance under *α* polarization is likely to be better than that under *π*-polarization, which provides theoretical support for the subsequent experimental results.

## 3 Device design and fabrication

**Figure 2:**
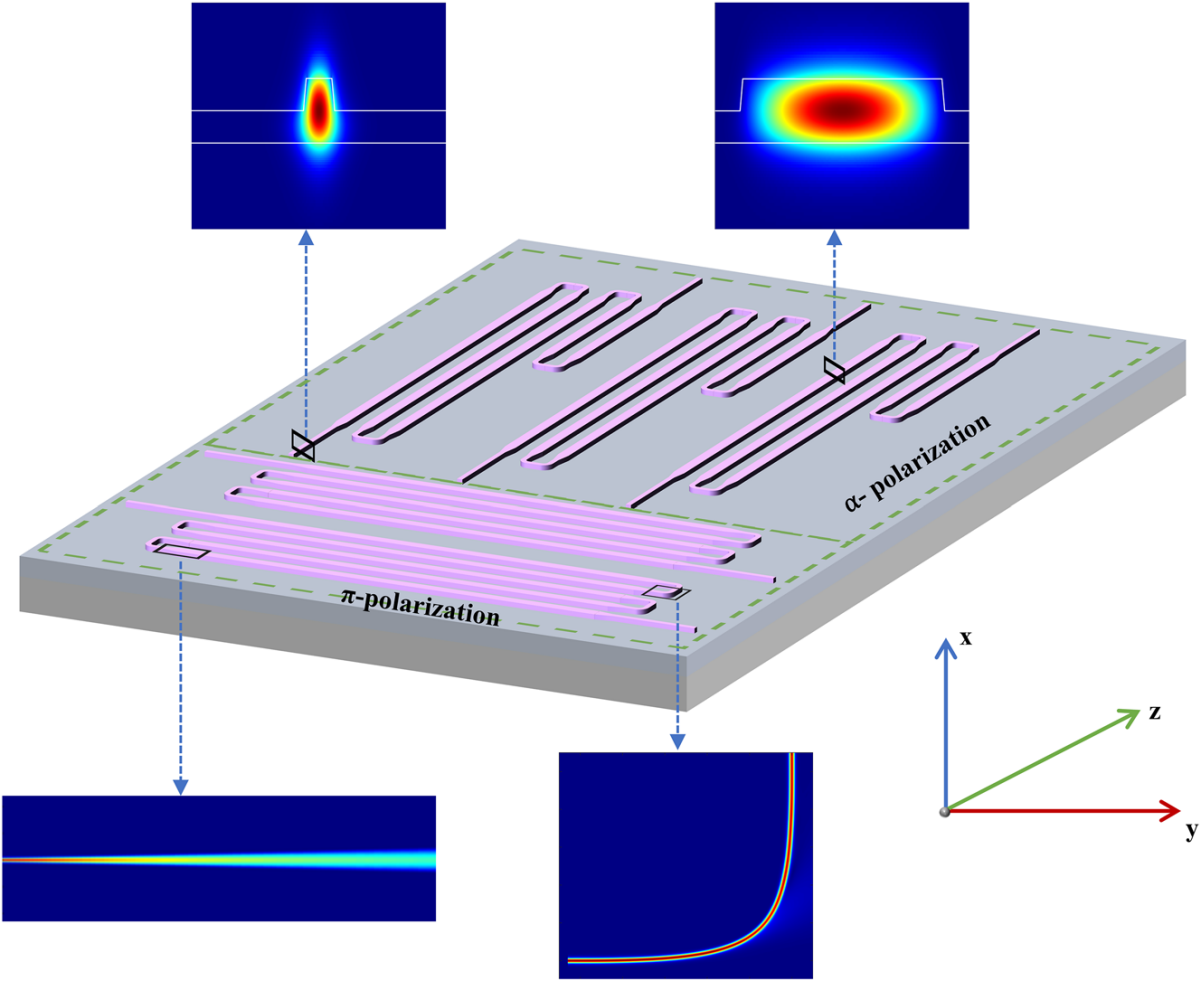
Schematic of the on-chip erbium-doped waveguide amplifier based on x-cut LNOI. The device features a folded waveguide geometry designed for TE_00_-mode propagation.

In this work, we designed two polarization-resolved EDWAs on x-cut Er:LNOI. The schematic of EDWAs is shown in Figure 2, which comprise a series of 9 μm-wide ridge waveguides as the gain region and 1 μm-wide ridge waveguides for TE_00_ mode input/output coupling, interconnected by 1,500 μm-long adiabatic tapers to preserve fundamental mode propagation without mode conversion or mixing. To ensure polarization stability and reduce the overall footprint, a folded waveguide geometry with compact Euler bends was implemented. The Euler bend waveguides have a width of 1 μm, which can simultaneously suppressing higher-order modes and reducing propagation losses. For centimeter-scale amplifiers, the contribution of bending loss can be considered negligible, or approximated as equivalent to the straight-waveguide propagation loss. Figure 2 also shows the simulated mode field profiles at different positions along the waveguide when the input is TE_00_ mode. The simulation results indicate that the optical field remains predominantly in the fundamental mode throughout the taper and Euler bend regions, with no significant excitation of higher-order modes or polarization conversion.

**Figure 3:**
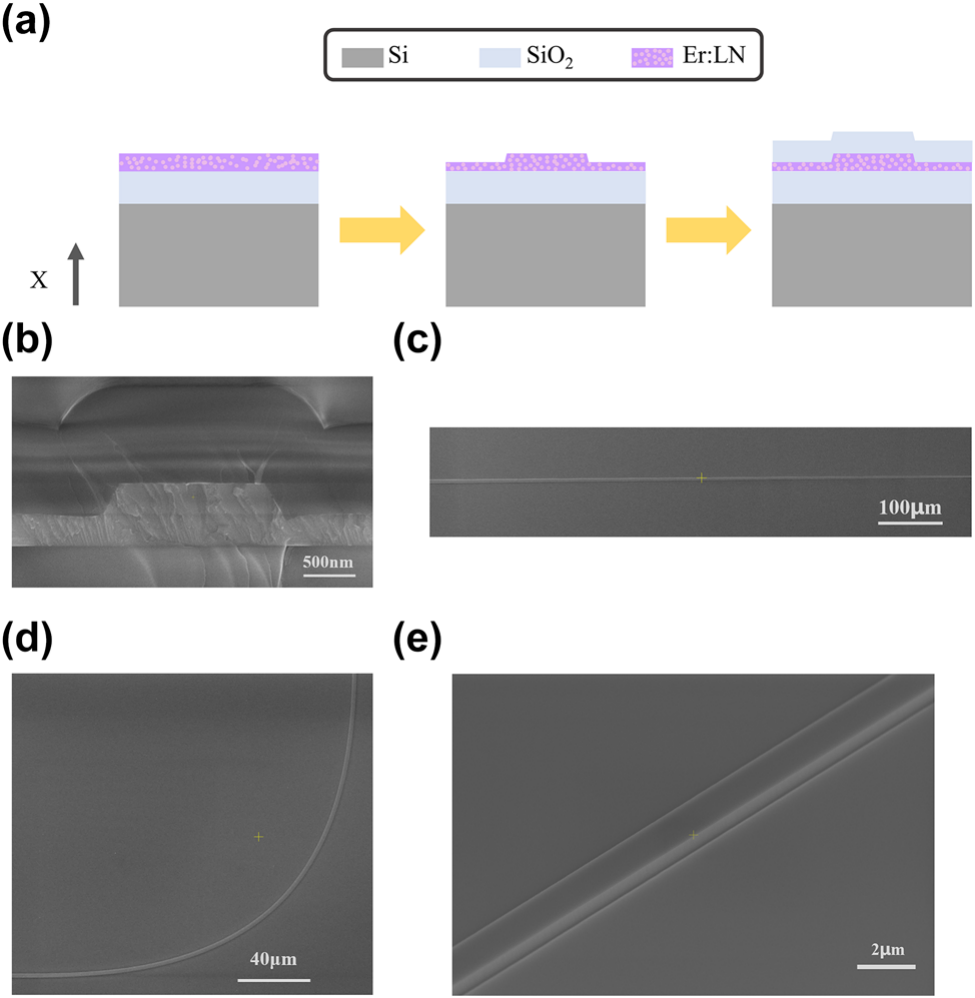
Fabrication and structural characterization of the EDWA. (a) Illustration of the fabrication of EDWA base on x-cut LNOI. (b, c, and d) SEM images of the fabricated EDWA at different locations, including the (b) cross sections of the straight waveguides, top views of the (c) adiabatic tapers and (d) Euler bends, (e) the sidewall of the waveguides.

Fabrication process of the EDWA starts from a high quality x-cut Er:LNOI wafer with an erbium doping concentration of 0.5 mol%, which is briefly explained in Figure 3(a). The wafer consists of a 600 nm-thick erbium-doped lithium niobate thin film, a 10 μm-thick SiO_2_ buffer layer, and a 500 μm-thick silicon substrate. First, electron-beam lithography (EBL) was employed to define the waveguide mask patterns. The mask patterns were then transferred into the Er:LNOI layer using inductively coupled plasma reactive ion etching (ICP-RIE), resulting in ridge waveguides with a 300 nm etching depth. Subsequently, the wafer was annealed at 500 °C to repair lattice damage induced during etching. A 1,000 nm-thick SiO_2_ cladding layer was then deposited over the waveguides via plasma-enhanced chemical vapor deposition (PECVD). Finally, we cleaved and polished both sides of the chip for edge coupling. Figure 3(b)–(e) shows scanning electron microscope (SEM) images of the EDWA at various locations, including straight waveguides, Euler-bend waveguides, adiabatic tapers, and waveguide sidewalls, all exhibiting smooth surfaces with approximately 65° inclination angles.

## 4 Result and discussion

### 4.1 Absorption loss under different polarizations

In order to accurately estimate the internal net gain of the EDWA, it is essential to properly evaluate the waveguide loss, mainly including the erbium absorption loss and propagation loss caused by waveguide sidewall roughness. Based on the preceding J–O analysis, TE modes propagating in x-cut Er:LNOI waveguides correspond to two distinct polarization states of the Er^3+^ ions. Due to the anisotropic crystal field, these polarizations exhibit different absorption and emission cross sections, which in turn lead to polarization-dependent absorption losses. The absorption loss $\alpha_{abs}\left(\lambda \right)$ due to Er^3+^ is related to the absorption cross section, as described by the following expression [38]:

(8) $$\alpha_{abs}\left(\lambda \right)=10{log}_{10}\left(e\right)\times \Gamma \left(\lambda \right)N_{d}\sigma_{abs}\left(\lambda \right)$$

where $\Gamma \left(\lambda \right)$ is the optical mode confinement factor in the Er:LNOI waveguide, *N*_*d*_ is the Er^3+^ concentration in Er:LNOI, and $\sigma_{abs}\left(\lambda \right)$ is the absorption cross section. The absorption loss described above represents the intrinsic absorption, defined under the assumption that all erbium ions remain in the ground state, which corresponds to the case where the signal power is near zero [29]. However, in practical measurements of absorption losses or gain characterization of EDWAs, the input signal power to the erbium-doped waveguides is never zero, and the actual absorption loss will be reduced due to excitation of erbium ions and corresponding depletion of the ground-state population. As a result, the total loss in the EDWA depends not only on the polarization state and signal wavelength but also on the input signal power.

**Figure 4:**
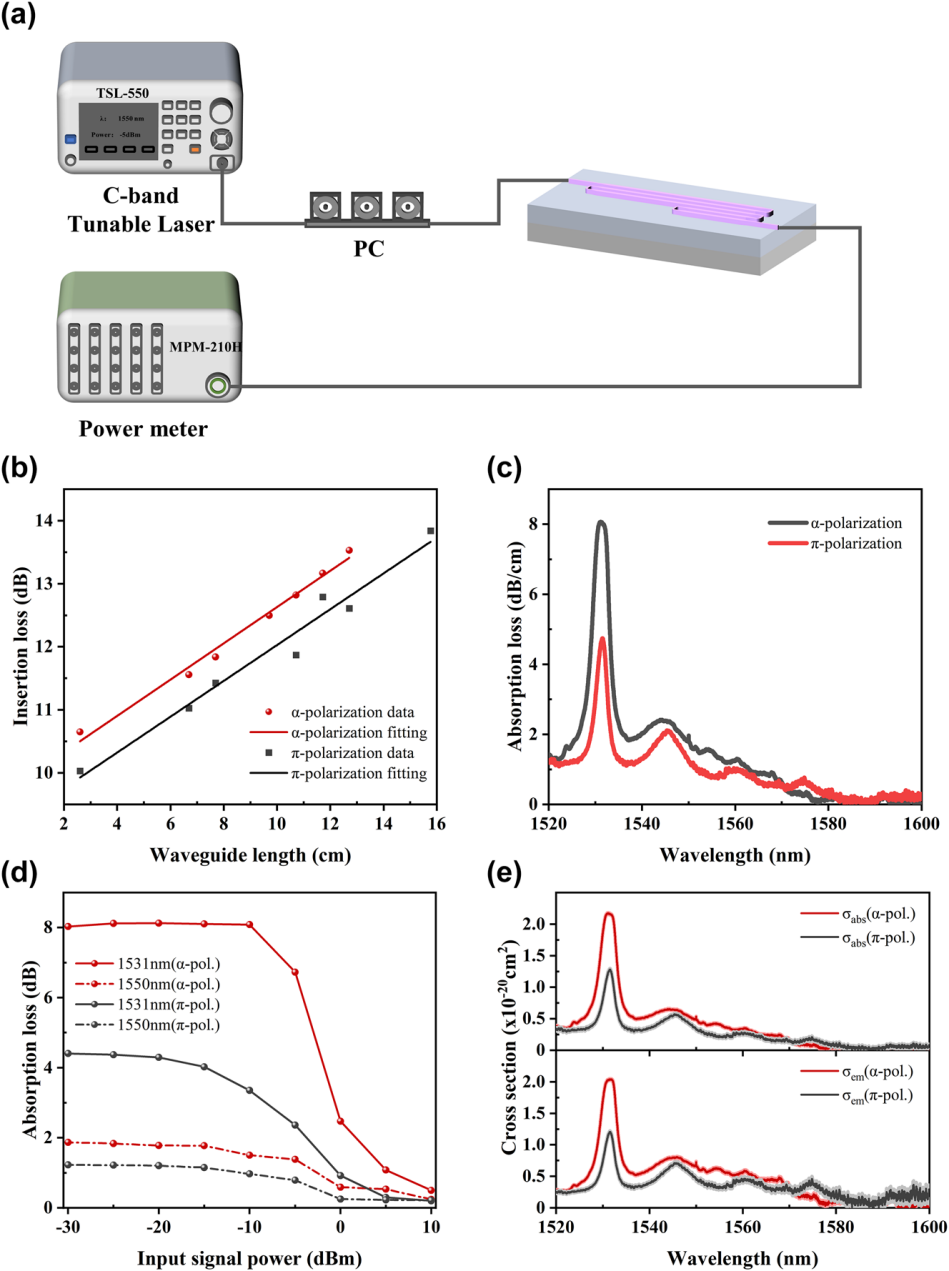
Characterization of polarization-dependent absorption loss in the EDWA. (a) Experimental setup for measuring the optical total loss. (b) Measured transmission losses at 1,580 nm for two polarizations with varying waveguide lengths, used to extract facet coupling and propagation losses. (c) Small-signal absorption loss spectra across the entire C-band for two EDWA devices with identical geometry but different TE mode polarization states. (d) The loss versus input power at different wavelengths for both polarization states. (e) Measured absorption and emission cross sections of two polarization states under 1,480 nm pumping, with the uncertainty envelopes derived from the cut-back fitting method.

We first characterized the coupling and propagation losses of EDWAs at 1,580 nm using the cut-back method, where the absorption of Er^3+^ ions is negligible [27], [34]. The experimental setup is illustrated in Figure 4(a), mainly consists of a C-band tunable laser source (TSL-550), a polarization controller, and an optical power meter (MPM-210H). This approach was adopted instead of using a ring resonator, since the x-cut Er:LNOI platform is polarization sensitive and a resonator cannot reliably distinguish the losses of *α*- and *π*-polarizations. To further minimize the potential influence of residual absorption, the source output power was set to 0 dBm during the measurement.

EDWAs with different lengths but the same polarization were measured on the same chip, ensuring that process variations between chips do not affect the results. Figure 4(b) shows the insertion loss versus waveguide length for both *α*- and *π*-polarizations. By linear fitting, the facet coupling losses were determined to be 4.88 ± 0.07 dB and 4.59 ± 0.12 dB per facet, and the propagation losses were 0.29 ± 0.01 dB/cm and 0.28 ± 0.02 dB/cm for the *α*- and *π*-polarizations, respectively. The small variations indicate that the extracted losses are highly consistent across waveguides of different lengths. Given that the designed device is capable of supporting both pump and signal wavelengths, the subsequent analysis approximates their facet coupling and propagation losses to be identical. For each polarization state, the fitted average values are employed in the calculations.

Then the absorption loss for each polarization can be obtained by subtracting the propagation loss from the on-chip total loss.

(9) $$\alpha_{abs}=\alpha_{total}-\alpha_{pro}$$

To determine the absorption loss under different polarization states, we performed C-band absorption loss measurement on two EDWAs with identical structures but different TE mode polarization orientations – corresponding to distinct erbium dipole alignments (*α*-polarization and *π*-polarization). We controlled the above setup via computer to form a rapid wavelength-sweeping measurement system for measuring the output power of the EDWAs across various signal wavelengths.

Figure 4(c) shows the absorption spectra for both polarization states with the source power set to −30 dBm. It is evident that the *α*-polarized EDWA exhibits significantly higher loss than the *π*-polarized one. Further comparison with the *σ*-polarization in z-cut devices shows that the absorption loss of the *α*-polarization at the peak wavelength of 1,531 nm is also substantially greater than that of both the *σ*- and *π*-polarizations.

To further study the power-dependent saturation behavior, we selected several representative wavelengths and measured the absorption loss as a function of input signal power, as presented in Figure 4(d). As the input power increases, the absorption loss gradually decreases, eventually saturating when approximately half of the erbium ions are excited, at which point the net absorption becomes zero. When the input signal power is as small as −30 dBm, the absorption loss no longer increases, approaching the intrinsic Er^3+^ absorption loss. The reason is that the absorption loss is proportional to the population of Er^3+^ remaining in the ground state when the absorption cross section is significant. As the signal power increases, more ions are excited, resulting in a reduced ground-state population and hence lower absorption loss. At 1,531 nm, the intrinsic absorption loss is 8.03 dB/cm for the *α*-polarization and 4.40 dB/cm for the *π*-polarization. At 1,550 nm, the values are 1.87 dB/cm and 1.22 dB/cm, respectively, indicating that the *α*-polarization consistently exhibits higher absorption loss than the *π*-polarization.

According to the McCumber’s theory, the absorption cross section and emission cross section obey the fundamental relation [35]:

(10) $$\sigma_{abs}=\sigma_{em}e^{\left(\frac{h\nu -\varepsilon }{kT}\right)}$$

where *T* is the temperature, *h* is the Planck’s constant, *k* is Boltzmann’s constant, and *ε* is the excitation energy related to temperature. In this study, *T* = 300 K and *ɛ* = 6,517 cm^−1^, which are consistent with the parameters reported in Ref. [35].

Therefore, based on the saturated absorption spectra of the two polarizations shown in Figure 4(c), we calculated the actual transition strength, absorption cross section, and emission cross section using Eqs. (6), (8), and (10), respectively.

Table 2 presents a comparison between the experimental transition strengths and the theoretical calculations. It can be observed that the transition intensity for *α*-polarization is larger than that for *π*-polarization, which is consistent with the theoretical trend. Meanwhile, the experimental values are generally lower than the theoretical ones, which may be attributed to the fact that the measured absorption spectrum does not fully cover the entire transition range. Nevertheless, the deviations between the experimental and theoretical results for both polarizations fall within the error range reported in the literature [30], indicating the validity and reliability of the results.

**Table 2:** Comparison of theoretical and experimental transition strengths under different polarization States. All values are in units of 10^−6^.

	*α*-polarization			*π*-polarization		
Transition	*f*_*α*,th_	*f*_*α*,meas_	Δ*f*	*f*_*π*,th_	*f*_*π*,meas_	Δ*f*
^4^I_15/2_ → ^4^I_13/2_	1.36	1.30	0.06	1.15	0.89	0.26

Figure 4(e) shows the absorption and emission cross sections over the entire C-band, together with their corresponding uncertainties. The uncertainties were evaluated based on the facet and propagation loss variances obtained from the cut-back fitting method. Using standard error propagation, it was found that the uncertainties of the absorption cross sections for both polarizations are below 1.9 × 10^−22^ cm^2^, while those of the emission cross sections are below 1.17 × 10^−21^ cm^2^. Particularly around 1,530 nm and 1,550 nm, these uncertainties are negligible.

Moreover, both the absorption and emission cross sections of the *α*-polarization are consistently larger than those of the *π*-polarization across the C-band. At the peak wavelength of 1,531 nm, the absorption and emission cross sections of the *α*-polarization are approximately 1.8 times those of the *π*-polarization, highlighting the significant polarization-dependent difference. In particular, at 1,550 nm, the *σ*_abs_ and *σ*_em_ of the *α*-polarization are 0.50 × 10^−20^ cm^2^ and 0.73 × 10^−20^ cm^2^, respectively, while those of the *π*-polarization show lower values of 0.33 × 10^−20^ cm^2^ and 0.48 × 10^−20^ cm^2^. The test results show that the absorption cross section at 1,580 nm is nearly zero, which further supports the validity of treating the loss at 1,580 nm as the propagation loss.

The theoretical prediction is consistent with our experimental results and also agrees with previous findings reported in Er^3+^-doped LiNbO_3_ bulk crystals [34], [39].

### 4.2 Signal enhancement and internal net gain

**Figure 5:**
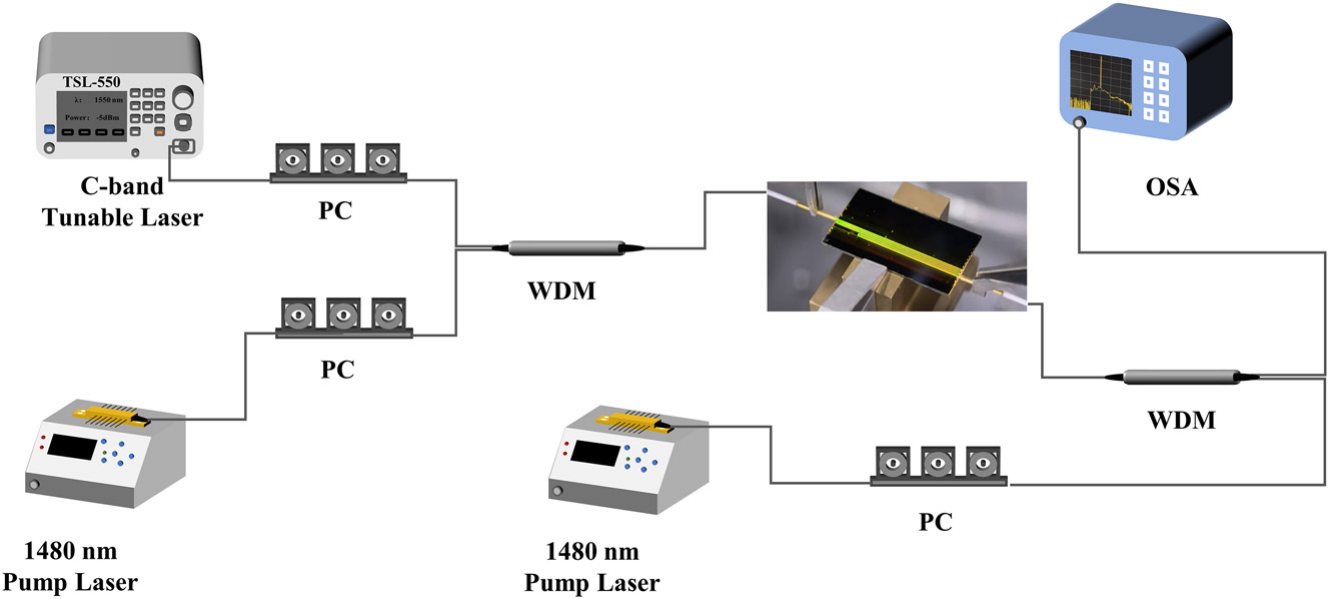
Experimental setup to characterize the gain of the EDWA. The digital camera photograph of the excited EDWA chip is shown in the center.

The measured differences in absorption and emission cross sections suggest that the gain performance of the EDWA should also be polarization-dependent. To verify this, we experimentally characterized the gain for both polarization states. First, we fabricated a series of *α*-polarized and *π*-polarized EDWAs with varying lengths on the same chip to evaluate the optimal gain length. The experimental setup is shown in Figure 5. The signal light is from a C-band tunable laser and the pump light is from a 1,480 nm laser diode. The pump light and signal light are combined via an input wavelength division multiplexer (WDM) and then coupled into the EDWA chip through the lensed fiber. The output WDM is used to inject backward pump light and extract the amplified signal light, which is then fed into an optical spectrum analyzer (OSA) for spectral measurement. Both signal light and pump light are adjusted to TE_00_ mode by polarization controllers (PC) to minimize propagation loss. Notably, under 1,480 nm pumping, the entire EDWA becomes uniformly illuminated by green upconversion fluorescence, visually verifying both the active erbium distribution and effective light confinement within the fabricated waveguides.

The signal enhancement *G*_se_ of the EDWA is quantified by measuring the output signal power difference between the pumped and unpumped states, expressed as:

$$G_{se}=10lg\frac{P_{on}}{P_{o\mathrm{ff}}}$$

where *P*_on_ and *P*_off_ represent the output signal power with and without pumping. This value provides an intuitive representation of the extent of signal enhancement under pumping conditions; however, the internal net gain should be regarded as the primary metric for evaluating actual amplifier performance.

The internal net gain is then obtained by subtracting the total loss from *G*_se_, expressed as:

$$G_{net}=G_{se}-\alpha_{total}=G_{se}-\alpha_{abs}-\alpha_{pro}$$

The net gain calculation method employed in this work is widely adopted for the characterization of erbium-doped lithium niobate waveguide amplifiers [23], [24], [25], [26], [27], [28], [29]. Unless otherwise specified, all gain values discussed herein refer to the on-chip internal net gain.

**Figure 6:**
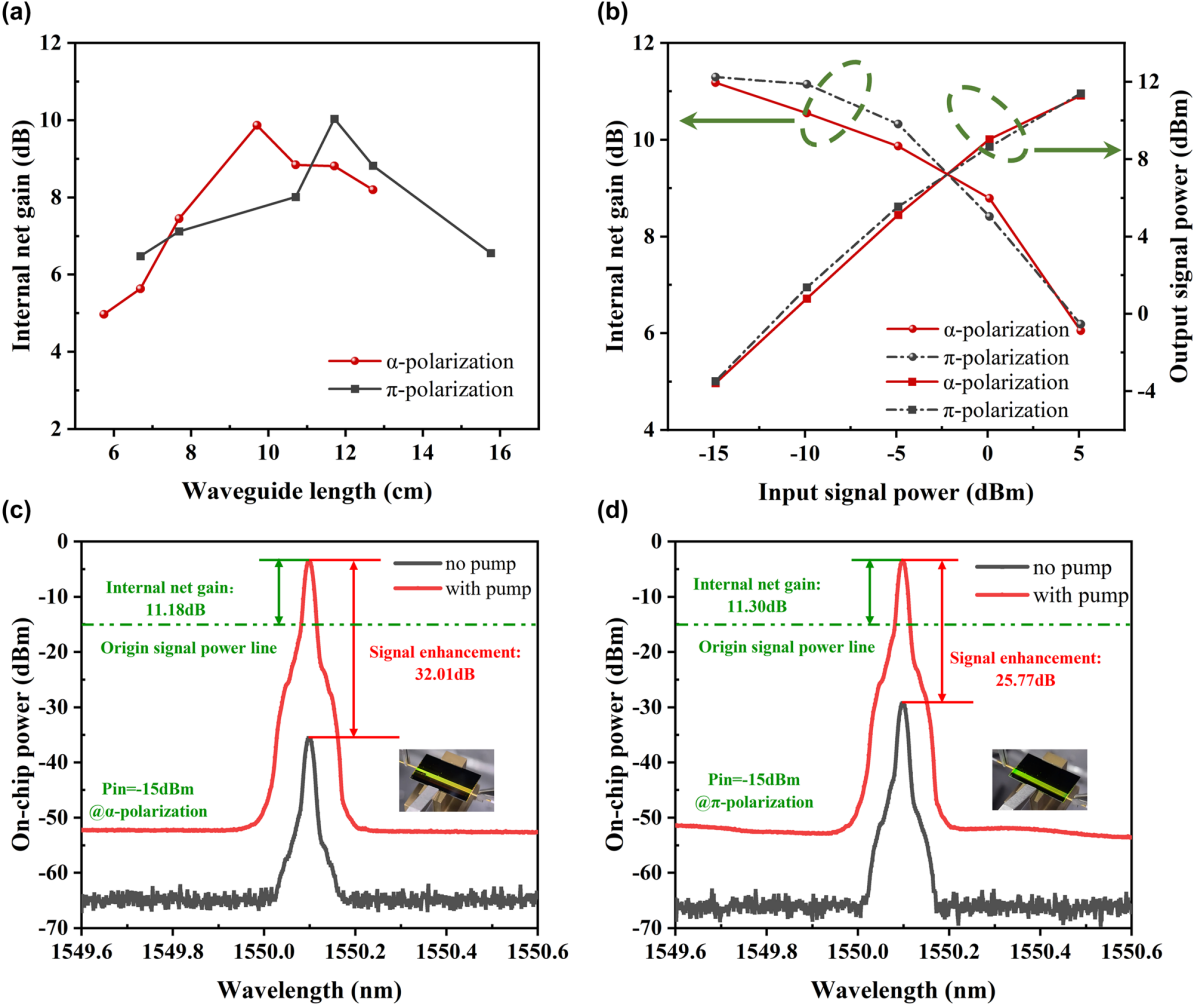
On-chip gain performance of the EDWA under different polarization states. (a) Internal net gain of *α*-polarized and *π*-polarized EDWAs with varying waveguide lengths under a fixed input signal power of −5 dBm at 1,550 nm. (b) Internal net gain and output signal power versus input signal power at 1,550 nm for both polarization states. (c) and (d) Spectra of the output signal power with and without pump at the maximum gain point for (c) *α*-polarization and (d) *π*-polarization. Insets show photos of the device with pump applied.

Figure 6(a) describes the internal net gain versus EDWA length for both polarization states under the same sufficient pump power excitation with a fixed on-chip input signal power of −5 dBm (all power values referenced to on-chip levels). The *α*-polarized and *π*-polarized configurations achieve nearly identical maximum net gains of 9.87 dB and 10.04 dB at their respective optimal waveguide lengths of 9.71 cm and 11.71 cm. Furthermore, we investigated the relationship between input signal power and gain characteristics. Figure 6(b) shows the internal net gain and output power as a function of input signal power for both polarization states at their respective optimal waveguide lengths. As the input signal power increases, the population inversion decreases, causing the internal net gain to gradually reduce. At first glance, the gain performance of the two polarization states appears similar, showing no significant polarization-dependent behavior. However, it’s worth noting that waveguide lengths for the *α*-polarization and *π*-polarization are different. As a result, their gain per unit length and signal enhancement are not directly equivalent. As shown in Figure 6(c) and (d), with an input signal power of approximately −15 dBm, the *α*-polarized EDWA exhibits 32.01 dB signal enhancement with 3.3 dB/cm unit gain, while the *π*-polarized EDWA reaches 25.77 dB signal enhancement with 2.2 dB/cm unit gain. According to the rate equation model, the net absorption rate of the pump and the net stimulated emission rate of the signal are both proportional to the absorption and emission cross sections. Therefore, *α*-polarization can achieve a higher population inversion within a shorter waveguide length, resulting in a larger gain coefficient than *π*-polarization and consequently a shorter optimum gain length.

To further investigate the evolution of signal gain with increasing pump power under different polarizations, we measured the gain evolution as a function of on-chip pump power under different input signal power levels at 1,550 nm and conducted model-based analysis using the experimental data.

**Figure 7:**
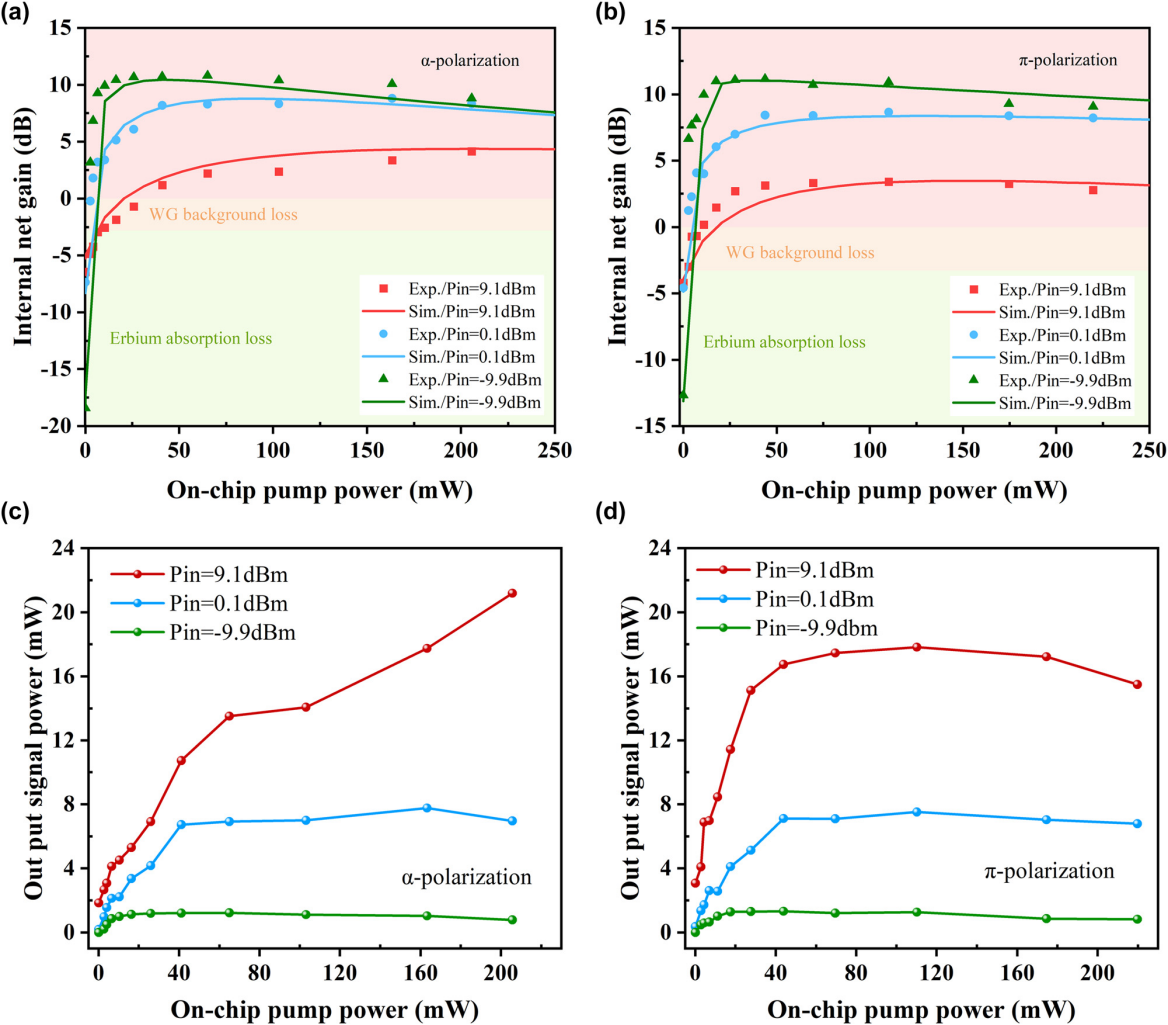
Amplifier performance under different pump power levels. Measured (scatters) and simulated (curves) internal net gain with respect to the on-chip pump power at three input signal powers for (a) *α*-polarization and (b) *π*-polarization; along with the measured on-chip output signal power for (c) *α*-polarization and (d) *π*-polarization.

As shown in Figure 7(a) and (b), the results show that under small-signal conditions, the *α* polarization, due to its larger stimulated absorption cross section and higher energy transfer up-conversion coefficient, tends to experience insufficient population inversion at high pump power, where the inversion becomes difficult to sustain and saturation occurs earlier, leading to a rapid gain roll-off. In contrast, under high input signal power, the strong signal significantly depletes the excited-state population, which makes the system more dependent on the pump. In this regime, although the *α* polarization still has a larger stimulated absorption cross section, it can maintain a relatively stable population inversion at high pump powers, and thus does not exhibit the same gain degradation observed in the small-signal regime.

For the *π*-polarization, due to its relatively smaller stimulated absorption cross section and lower energy transfer up-conversion coefficient, it can maintain a more stable population inversion under small-signal conditions, resulting in smoother gain growth at low to moderate pump powers. However, when the input signal power is high, the signal significantly depletes the inversion, making the system more reliant on strong pumping to replenish the excited-state population. Although the pump power increases, the *π*-polarization exhibits a relatively smaller absorption cross section for the pump light, and the longer propagation length further reduces the effective pumping efficiency. As a result, even under high pump powers, it is difficult to maintain sufficient population inversion, leading to gain saturation and eventual decline.

Figure 7(c) and (d) shows the on-chip output power as a function of on-chip pump power. At an input signal power of 9.1 dBm, the *α*-polarized device achieves a maximum on-chip output power of 21.18 mW, and the *π*-polarized counterpart reaches 17.82 mW. However, based on the observed trend of the output power curves, it can be expected that under increased pump power, *α*-polarization still has the potential to further enhance output power, exhibiting a higher saturation gain limit. This higher saturation gain limit and pump demand are attributed to a faster inversion depletion rate in the *α*-polarization, resulting from its larger absolute absorption cross section coupled with stronger excited-state absorption and upconversion.

## 5 Conclusions

### 5.1 Polarization-dependent gain performance and mechanism

The measured absorption and emission cross sections exhibit a pronounced disparity between *α*- and *π*-polarizations. At the peak wavelength of 1,531 nm, the cross sections for *α*-polarization are 1.8 times greater than those for *π*-polarization. More notably, at the standard telecommunication wavelength of 1,550 nm, the absorption and emission cross sections were quantified as 0.50 × 10^−20^ cm^2^ and 0.73 × 10^−20^ cm^2^ for *α*-polarization, compared to 0.33 × 10^−20^ cm^2^ and 0.48 × 10^−20^ cm^2^ for *π*-polarization, respectively.

This substantial difference in cross sections directly leads to divergent amplifier gain performance. The higher cross sections of the *α*-polarization result in a higher gain coefficient of 3.3 dB/cm, allowing it to achieve optimal amplification in a shorter device length of 9.71 cm. In contrast, the *π*-polarization, with its lower gain coefficient of 2.2 dB/cm, requires a longer interaction length of 11.71 cm to attain comparable gain levels. Consequently, under high input signal power conditions, the *α*-polarized amplifier delivers a superior maximum unsaturated output power of 21.18 mW, outperforming the 17.82 mW achieved by the *π*-polarized device.

These performance discrepancies originate fundamentally from the crystalline anisotropy of the x-cut LNOI substrate, which strongly influences the transition strengths of the Er^3+^ ions. Judd–Ofelt theoretical calculations confirm that the transition strength for *α*-polarization is significantly higher than that for *π*-polarization. This directly predicts the larger cross sections observed experimentally and provides a rigorous physical explanation for the enhanced gain and output performance of the *α*-polarization across the C-band.

### 5.2 Comparison with conventional z-cut platform

To evaluate the implications of our platform choice, we compare the performance of our x-cut amplifier with the well-established z-cut platform in Table 3. The comparison reveals a clear trade-off: while z-cut EDWAs generally yield higher absolute net gain and output power [24], [25], [26], [28], [29], their performance is largely isotropic. In contrast, the x-cut platform provides a decisive new degree of freedom – polarization-dependent gain – as demonstrated in this work. More importantly, it offers superior electro-optic (EO) and nonlinear optic (NLO) compatibility due to facile access to the dominant r_33_ and d_33_ coefficients, which is a fundamental requirement for future densely integrated photonic circuits.

**Table 3:** Performance comparison between conventional z-cut and this work on x-cut Er:LNOI waveguide amplifiers^a^.

Ref.	Orientation	Polarization^b^	Device length (cm)	Pump wavelength (nm)	Pump power (mW)	Signal wavelength (nm)	Input signal power (dBm)	Internal net gain (dB)^c^	Output power (dBm)	EO/NLO compatibility	Implication/trade-off
[24]	Z-cut	TE (*σ*-pol)	3.6	980	40	1,530	−47.07	18	−29.07	Poor (relies on r_13_)	Z-cut platforms generally achieve higher absolute gain
					26	1,550	−38.38	7.5	−30.88		
[25]	Z-cut	TE (*σ*-pol)	2.58	1,484	17.31	1,531.6	−50	16	−34	Poor (relies on r_13_)	X-cut offers polarization-sensitive gain, a key feature for advanced control
					18.54	1,550	−50	2.66	−47.34		
[26]	Z-cut	TE (*σ*-pol)	10	976	63	1,532	−22	20	−2	Poor (relies on r_13_)	*α*-pol requires a shorter device length for peak performance
[28]	Z-cut	TE (*σ*-pol)	7	1,486	210	1,550	4.5	16	20.5	Poor (relies on r_13_)	*π*-pol requires significantly lower pump power
					63	1,531.5	−33	27.6	−5.4		
[29]	Z-cut	TE (*σ*-pol)	10	1,484	N/A	1,531	−15	22.26	7.26	Poor (relies on r_13_)	Z-cut enables higher output power levels
					120	1,550	9	7.65	16.65		
This work	X-cut	TE (*α*-pol)	9.71	1,480	65	1,550	−15	11.18	−3.82	Excellent (full r_33_ acess)	X-cut is superior for monolithic integration with modulators & wavelength converters
					206	1,550	9.1	4.16	13.26		
This work	X-cut	TE (*π*-pol)	11.71	1,480	65	1,550	−15	11.30	−3.7	Excellent (full r_33_ acess)	
					110	1,550	9.1	3.41	12.51		

^a^All results are approximate values obtained from the reported literature. ^b^The *α*-, *σ*-, and *π*-polarizations refer to the Er^3+^ transition orientations determined by the electric-field and propagation directions relative to the optical axis. ^c^All gain values correspond to the total on-chip internal net gain, excluding coupling losses.

### 5.3 Advancements beyond prior work on x-cut LNOI

Having contextualized the x-cut platform against the z-cut standard, we now highlight the specific advancements of our work within the emerging field of x-cut Er:LNOI amplifiers.

As summarized in Table 4, pioneering studies by Luo et al. [40] and Xue et al. [27] successfully demonstrated the feasibility of achieving optical gain on this platform. However, their analyses were not designed to resolve the fundamental polarization-dependent characteristics inherent to the x-cut orientation.

**Table 4:** Comparison with prior work on x-cut Er:LNOI waveguide amplifiers.

Ref.	Gain reported	Polarization resolved	Key achievement	Advancement of this work
[23]	Yes	No	First demonstration of an EDWA on x-cut LNOI, proving feasibility	Provides the first comprehensive polarization-resolved analysis of gain, cross sections, and loss
[27]	Yes	No	First amplifier fabricated on um-thick x-cut LNOI, demonstrating higher gain	First quantification of polarization-dependent gain coefficients (3.3 vs 2.2 dB/cm)
This work	Yes	Yes	Systematic characterization of polarization-dependent spectral and amplification properties	Supplies key data and design rules for developing polarization-aware integrated functional devices

Our work provides the first comprehensive, polarization-resolved analysis of transition strength, gain, cross sections, and saturation properties on this platform. This dataset provides the indispensable foundation for designing sophisticated, polarization-aware functional devices on this platform.

### 5.4 Conclusion and outlook

In summary, this work systematically investigated the polarization-dependent gain characteristics of EDWAs on the x-cut LNOI platform. Guided by J–O theory, we analyzed the variations in Er^3+^ transition strength and rate-equation parameters for different polarizations, revealing the underlying mechanism of how crystal orientation and polarization state affect device performance. Experimental validation confirmed significant differences in absorption/emission cross sections and gain coefficients between *α*- and *π*-polarizations, leading to distinct trends in optimal gain length, gain saturation, and output performance. A maximum unsaturated output power of 21.18 mW is achieved under 1,480 nm pumping in a 9.71 cm-long *α*-polarized EDWA, indicating the potential for even higher output power with increased pump power. This study provides the first comprehensive insight into polarization-selective amplification in x-cut Er:LNOI and establishes both theoretical and experimental foundations for designing efficient, fully integrated EDWAs, paving the way for large-scale photonic integration on a unified photonic platform.
